# Serum proBDNF/BDNF and response to fluvoxamine in drug-naïve first-episode major depressive disorder patients

**DOI:** 10.1186/1744-859X-13-19

**Published:** 2014-07-09

**Authors:** Reiji Yoshimura, Taro Kishi, Hikaru Hori, Kiyokazu Atake, Asuka Katsuki, Wakako Nakano-Umene, Atsuko Ikenouchi-Sugita, Nakao Iwata, Jun Nakamura

**Affiliations:** 1 Department of Psychiatry, University of Occupational and Environmental Health, Kitakyushu 807-8555, Japan; 2 Department of Psychiatry, Fujita Health University, Toyoake 470-1192, Japan

**Keywords:** BDNF, proBDNF, Major depressive disorder, Serum, Fluvoxamine

## Abstract

**Background:**

We investigated the association between serum proBDNF, a precursor of brain-derived neurotrophic factor (BDNF), and response to fluvoxamine in patients with major depressive disorder (MDD) using the Diagnostic and Statistical Manual of Mental Disorders, Fourth Edition, Text Revision (DSM-IV-TR): physically healthy and free of current alcohol or drug abuse, comorbid anxiety, or personality disorders.

**Methods:**

Fifty-one patients with MDD (M/F, 19:32; age, 38 ± 19 years) and 51 healthy controls (M/F, 22:29; age, 34 ± 17 years) were studied using DSM-IV-TR: physically healthy and free of current alcohol or drug abuse, comorbid anxiety, or personality disorders. Serum levels of proBDNF and MDNF were measured by sandwich enzyme-linked immunosorbent assay (ELISA).

**Results:**

Serum mature BDNF levels in the MDD patients were significantly lower than those in the healthy controls (*t* = 3.046, *p* = 0.0018). On the other hand, no difference was found in serum proBDNF between the MDD patients and the healthy controls (*t* = −0.979, *p* = 0.833). A trend of negative correlation was found between baseline serum BDNF and baseline scores of the 17 items of the Hamilton Rating Scale for Depression (HAMD17) (*r* = −0.183, *p* = 0.071). No correlation was however found between HAMD17 scores and proBDNF at baseline (*r* = 0.092, *p* = 0.421). Furthermore, no correlation was observed between baseline HAMD17 scores and baseline proBDNF/BDNF (*r* = −0.130, *p* = 0.190). No changes were observed in serum levels of proBDNF and BDNF during the treatment periods.

**Conclusions:**

These results suggest that there is no association between serum proBDNF/BDNF and fluvoxamine response in MDD patients at least within 4 weeks of the treatment.

## Background

Major depressive disorder (MDD) is a common and major psychiatric disorder that affects as many as about 20% of individuals within their lifetime [1-3]. A wide variety of pharmaceuticals are available for treating depression, including tricyclic antidepressants, monoamine oxidase inhibitors, and selective serotonin reuptake inhibitors (SSRIs). Fluvoxamine is an SSRI that is widely used for treatment of depression and other psychiatric disorders and has been suggested to have early effects when used as an antidepressant drug [4,5]. In addition, the results of a meta-analysis have shown that significant improvements in Hamilton Rating Scale for Depression (HAMD) scores achieved in the first few weeks were maintained after 6 weeks of treatment [6]. Results of a recent meta-analysis also suggest that treatment with fluvoxamine leads to symptomatic improvements in patients with MDD by the end of the first week of use [6].

Mature brain-derived neurotrophic factor (BDNF) is initially synthesized as a precursor protein. ProBDNF is converted to BDNF by extracellular proteases such as matrix metalloproteinase-9 (MMP-9). BDNF is biologically active. In contrast, proBDNF, which localizes intracellularly, serves as an inactive precursor. In short, new evidence shows that proBDNF and BDNF elicit opposing effects via the neurotrophin receptor p75 (p75NTR) and tropomyosin-related kinase B (TrkB) receptors, respectively, and that both proBDNF and mature BDNF play important roles in several physiological functions for neurons, which might be related to the pathology of psychiatric disorders such as mood disorders and schizophrenia [7-9]. Sen et al. [10] first performed a meta-analysis and demonstrated that serum BDNF levels are abnormally low in patients suffering from major depressive disorder and that the BDNF levels are elevated following a course of antidepressant treatment. Although the relationship of our findings to the pathophysiology of depression and the mechanism of drug action remains to be determined, the measure may have potential use as a biomarker for psychiatric disorders or as a predictor of antidepressant efficacy [10,11]. Recently, Yoshida et al. [12] reported that it was initially thought that only secreted mature BDNF was biologically active and that proBDNF, which localizes intracellularly, served as an inactive precursor. However, new evidence shows that proBDNF and BDNF elicit opposing effects via the p75NTR and TrkB receptors, respectively, and that both proBDNF and BDNF play important roles in several physiological functions [8,12]. In recent decades, the role of BDNF in first-episode major depressive disorder MDD patients has received intensive attention. However, the relationship between proBDNF and MDD has not been clearly elucidated. We hypothesized that (1) serum levels of BDNF, proBDNF, and proBDNF/BDNF are different between MDD patients and healthy controls, (2) fluvoxamine decreases serum proBDNF level and proBDNF/BDNF ratio and increases serum BDNF level, and (3) the plasma level of fluvoxamine is related to serum levels of BDNF and the HAMD17 scores.

This study aimed to determine whether serum levels of proBDNF/BDNF were different between MDD patients and the healthy controls. We also examined serum levels of proBDNF/BDNF between the responders and the nonresponders to fluvoxamine in patients with first-episode MDD. In addition, we also investigated longitudinal changes in proBDNF and BDNF in MDD patients treated with fluvoxamine before and after the treatment.

To the best of our knowledge, this is the first study investigating serum proBDNF/BDNF and response to fluvoxamine.

## Materials and methods

### Participants

Fifty-one drug-naïve and first-episode patients with MDD were studied. In the MDD group, 19 were males and 32 were females, ranging in age from 29 to 71 (mean ± standard deviation (SD), 38 ± 19) years. In 51 cases of the healthy control (HC) group, 22 males and 29 females, ranging in age from 24 to 68 (mean ± SD, 35 ± 16) years, were enrolled in the present study. Prior to the commencement of the study, all subjects provided written informed consent, after receiving a full explanation of the study as well as any potential risks and benefits of study participation. The study was approved by the Ethics Committee of the University of Occupational and Environmental Health and performed in accordance with the Declaration of Helsinki II. The demographics of the participants are shown in Table 1, ranging in age from 29 to 71 (mean ± SD, 38 ± 19) years. All patients fulfilled the MDD criteria using the Diagnostic and Statistical Manual of Mental Disorders, Fourth Edition, Text Revision (DSM-IV-TR): physically healthy and free of current alcohol or drug abuse, comorbid anxiety, or personality disorders. We defined the responders as those whose scores of the 17 items of the Hamilton Rating Scale for Depression (HAMD17) decreased 50% or more. All patients consented to participate after having been informed of the study's purpose. Benzodiazepines were the only hypnotics permitted, and their dosages were kept constant throughout the study period. The dosages of fluvoxamine varied among patients and were not fixed for ethical reasons.

**Table 1:** Demographics of participants

	**Values**
Age (years)	38 (19)
Female (%)	62
Daily dose at week 4 (max) (mg)	103 (38)
DUP (months)	2.1 (0.9)
HAMD17 (baseline)	19.3 (2.8)

### Assessment of clinical variables

Depression was assessed using the 17 items of the Structured Interview Guide for the Hamilton Depression Rating Scale (SIGH-D) by an experienced psychiatrist (R.Y.).

### Serum proBDNF and BDNF assay

Blood was drawn at 9:00 a.m. Serum levels of BDNF and proBDNF were measured in duplicate using the human proBDNF enzyme-linked immunosorbent assay (ELISA) kit SK00572-06 (Adipo Bioscience, Santa Clara, CA, USA) and the human matureBDNF ELISA kit SK00572-01(Adipo Bioscience, Santa Clara, CA, USA). All experiments were performed in duplicate. Protocols were performed according to the manufacturer's instructions. In short, 96-well microplates were coated with anti-BDNF monoclonal antibody and incubated at 4°C for 18 h. The plates were incubated in a blocking buffer for 1 h at room temperature. The samples were diluted 100-fold with an assay buffer, and BDNF standards were kept at room temperature with horizontal shaking for 2 h and then washed with the appropriate washing buffer. The plates were incubated with antihuman BDNF polyclonal antibody at room temperature for 2 h and washed with the washing buffer. Then, they were incubated with anti-IgY antibody conjugated to horseradish peroxidase for 1 h at room temperature and incubated again in peroxidase substrate and tetramethylbenzidine solution to induce a color reaction. The reaction was stopped with 1 mol/L hydrochloric acid. The absorbance at 450 nm was measured with an Emax automated microplate reader (Molecular Devices, Chuo-ku, Japan). Measurements were performed in duplicate.

### Plasma fluvoxamine assay

The plasma fluvoxamine level was measured using high-performance liquid chromatography according to the method we previously described [13]. In brief, 1 mL of plasma alkalized with 500 μL of 2 M sodium hydrogen carbonate was extracted by hexane (10 mL) after the addition of the internal standard (clomipramine). Shaken horizontally for 20 min and then centrifuged at 2,000 *g* for 10 min, the upper organic layer was removed and dried under N_2_. After being dissolved in 200 μL of mobile phase, a 50-μL aliquot of the final preparation was subjected to HPLC. All experiments were performed in duplicate.

### Statistical analyses

Student's *t* test was used to compare two groups (serum levels of proBDNF and BDNF; healthy control vs. MDD). Serum levels of proBDNF and BDNF and plasma fluvoxamine concentrations were measured, and correlations with clinical variables were performed using Peason's correlation. One-way ANOVA was used regarding the time course of proBDNF and BDNF. Power analysis was performed in BDNF (0 week) × HAMD17 (0 week) and BDNF (healthy control, 0 week) × BDNF (MDD, 0 week). A significant value of *p* < 0.05 was judged as statistically significant. All analyses were carried out using SPSS version 19.0 (SPSS Inc, Chicago, IL, USA).

## Results

**Figure 1:**
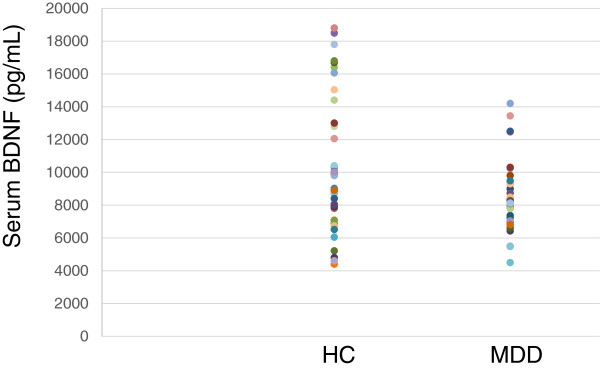
**The HAMD17 scores and serum proBDNF.** Red line shows the mean of value.

**Figure 2:**
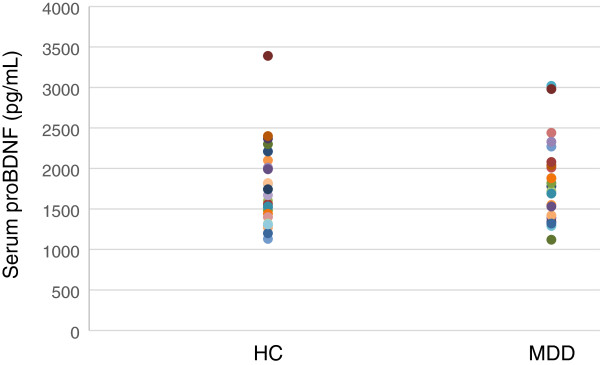
**Serum proBDNF change during treatment with fluvoxamine.** Red line shows the mean of value.

**Figure 3:**
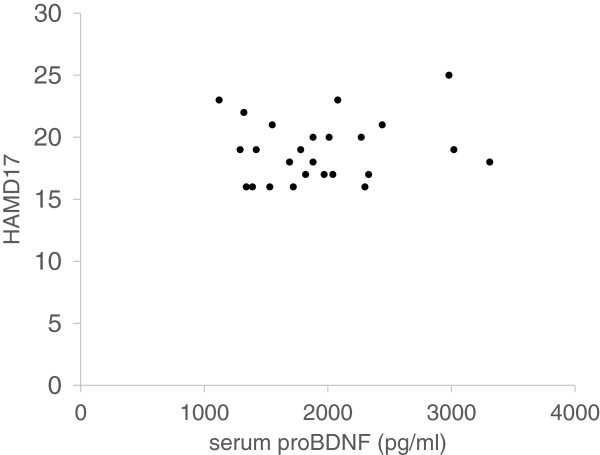
Serum proBDNF in the responders' and the nonresponders' treatment with fluvoxamine.

**Figure 4:**
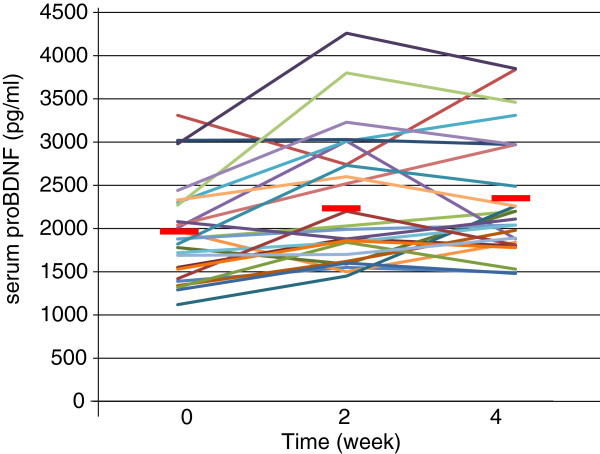
**The HAMD17 scores and serum BDNF.** Red line shows the mean of value.

**Figure 5:**
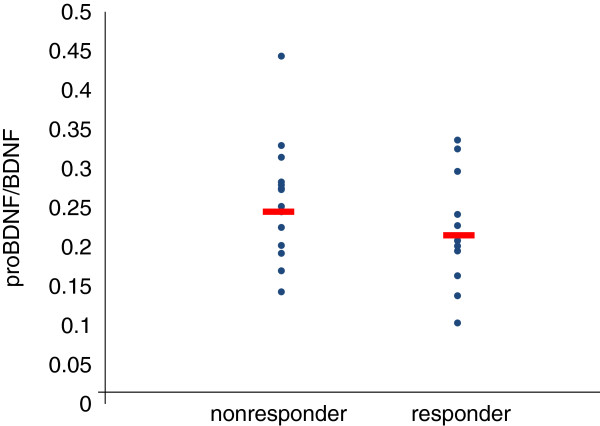
Serum BDNF change during treatment with fluvoxamine.

**Figure 6:**
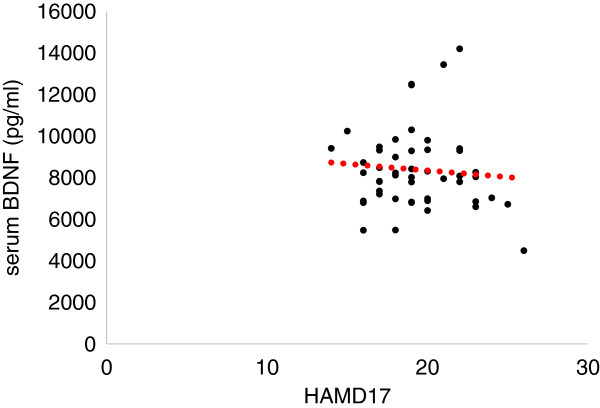
**Serum BDNF in the responders' and the nonresponders' treatment with fluvoxamine.** Red dot line shows regression line.

**Figure 7:**
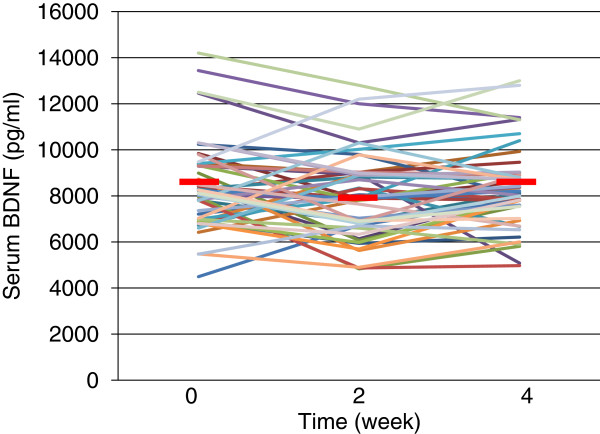
**Serum BDNF change during treatment with fluvoxamine.** Red line shows the mean of value.

**Figure 8:**
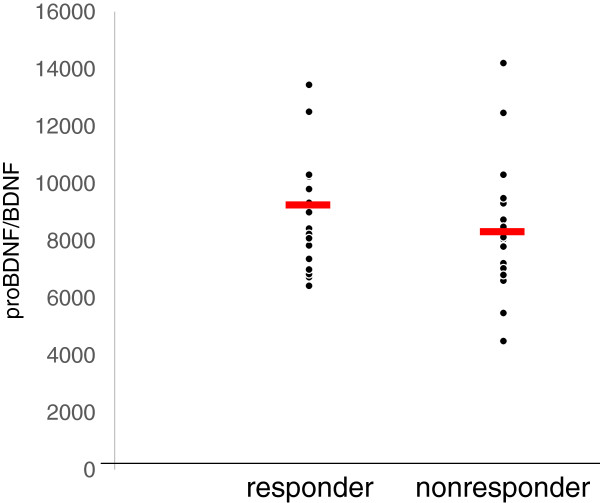
**The proBDNF/BDNF ratio between the responders and the nonresponders.** Red line shows the mean of value.

**Figure 9:**
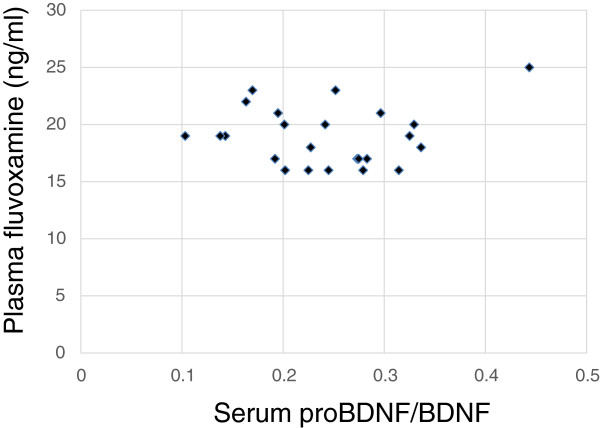
The proBDNF/BDNF ratio and plasma fluvoxamine concentration.

**Figure 10:**
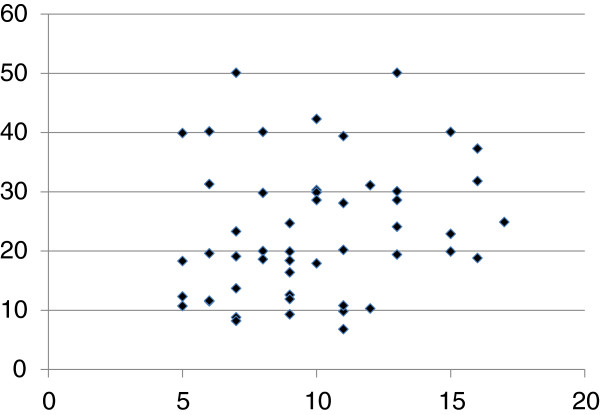
The changes in the HAMD17 (0–4 weeks) and plasma fluvoxamine concentration at week 4.

**Figure 11:**
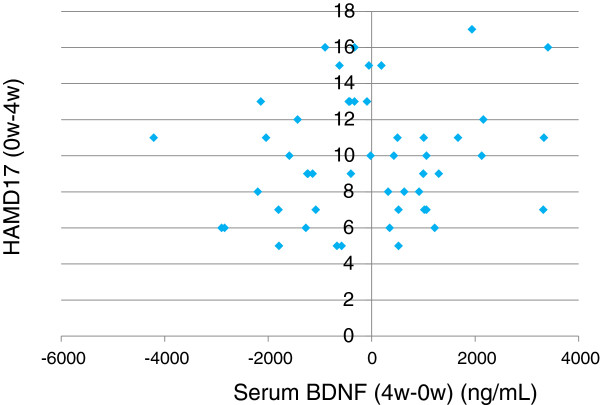
The changes in the HAMD17 (0–4 weeks) and plasma concentration at week 4.

The demographics of the participants are shown in Table 1. Twenty-five of 51 (49%) MDD patients responded to fluvoxamine at least within 4 weeks. Nine of 51 (18%) MDD patients had remission. Serum BDNF of all subjects could be measured. Serum proBDNF of 32 of 51 HC (63%) and 25 of 51 MDD patients (49%) could be assayed. Serum BDNF levels in MDD were significantly lower than those in HC (*t* = 3.046, *p* = 0.0018, 1-β = 82.3%) (Figure 1). On the other hand, no difference was found in serum proBDNF between the MDD patients and the HC (*t* = −0.979, *p* = 0.833) (Figure 2). Twenty-four of 51 MDD patients (47%) responded to fluvoxamine treatment at least within 4 weeks. No difference was found in baseline proBDNF between responders and nonresponders (*t* = 1.837, *p* = 0.073). No difference was also found in baseline BDNF between responders and nonresponders (*t* = 1.19, *p* = 0.23). A trend for negative correlation was found between baseline serum BDNF and baseline HAMD17 scores (*r* = −0.183, *p* = 0.071) (Figure 3). No correlation was however found between the HAMD17 scores and proBDNF at baseline (*r* = 0.092, *p* = 0.421) (Figure 4). No difference was found between serum levels of proBDNF (*r* = 0.090, *p* = 0.336) (Figure 5) and BDNF (*r* = −0.084, *p* = 0.730) (Figure 6) at week 0 in MDD patients. No changes were observed in serum levels of proBDNF at baseline, 2 weeks, and 4 weeks after administrating fluvoxamine (*F* = 2.580, *p* = 0.080) (Figure 7). No changes were also observed in serum levels of BDNF at baseline, 2 weeks, and 4 weeks after administrating fluvoxamine (*F* = 0.579, *p* = 0.561) (Figure 8). Furthermore, no correlation was observed between baseline HAMD17 scores and baseline proBDNF/BDNF in MDD patients (*r* = −0.130, *p* = 0.190) (Figure 9). No correlation was also found between plasma fluvoxamine levels at week 4 and the changes in HAMD17 scores (*r* = 0.211, *p* = 1.514) (Figure 10). No correlation was found between plasma fluvoxamine levels at 4 weeks and the changes in serum BDNF levels (from 0 to 4 weeks) (*r* = 0.117, *p* = 0.691) (Figure 11).

## Discussion

Recent meta-analyses demonstrated that mature BDNF levels in serum in patients with MDD were decreased compared to those in healthy controls. The result in the present study regarding serum BDNF confirms the recent meta-analyses [10,14,15]. Low serum mature BDNF levels increased over the course of antidepressant treatment [10,14,15]. We have previously reported that a significant correlation was found between the HAMD17 score and serum BDNF levels before pharmacotherapy [16]. In the present study, we reconfirmed our previous finding. A correlation was not however observed between serum proBDNF levels and the HAMD17 scores before starting fluvoxamine. In addition, there was no relationship between serum levels of proBDNF/BDNF and HAMD17. Taking these findings into account, the BDNF level, but not proBDNF and proBDNF/BDNF, reflects the severity of MDD. Moreover, no correlation was observed between serum fluvoxamine levels and serum levels of proBDNF/BDNF. The result in the present study suggests that the plasma fluvoxamine level was not independent of proBDNF/BDNF in MDD patients after fluvoxamine treatment. In addition, serum levels of BDNF and proBDNF/BDNF did not change at least 4 weeks after fluvoxamine administration. However, serum proBDNF increased during fluvoxamine treatment but did not reach the statistically significant level. Taking these findings into account, our hypothesis was not confirmed. In other words, the influence of fluvoxamine on serum levels of proBDNF, BDNF, and proBDNF/BDNF is complicated. Another interpretation is that 4 weeks is not enough to alter the dynamics of proBDNF and BDNF. Zhou et al. [17] reported that protein and serum levels of proBDNF were higher in MDD than in healthy control subjects while BDNF levels were lower. The authors also demonstrated that the levels of BDNF and proBDNF negatively and positively correlated with major depression severity, respectively. These results suggest that the balance between proBDNF and BDNF is disturbed in MDD. Sodersten et al. [18] recently reported a very interesting finding using two independent cohort studies (Sahlgrenska cohort and Karolinska cohort). The authors found that serum MDNF, proBDNF, the ratio of BDNF/proBDNF, and interaction with MMP-9 were different between patients with bipolar disorders and healthy controls. The function of proBDNF however remains precisely elucidated.

Furthermore, there is little information about the role of serum proBDNF. We know that a controversy exists about the relationship between brain BDNF and peripheral BDNF. A recent study reported that circulating BDNF revealed a positive correlation with hippocampal BDNF, which reinforces the relevance to identify a potentially useful therapeutic biomarker [19].

No correlation was found between the changes in the HAMD17 scores and plasma fluvoxamine levels, which indicates that the effect of plasma fluvoxamine levels is independent of an individual's depressive clinical efficacy in fluvoxamine. In other words, the pharmacodynamic factors of each patient might also be involved in the effects of fluvoxamine. We should consider various factors for predicting the treatment response, and it could be more complicated in the fluvoxamine response. The present study had several limitations: (i) small samples, (ii) assaying serum proBDNF was tricky and the detection rate of serum proBDNF was very low using the ELISA kit, and (iii) we did not measure MMP-9 levels. Thus, we are undergoing reconfirmation of these preliminary results using another ELISA kit or Western blotting method.

## Conclusions

We reconfirmed that serum levels of BDNF, but not proBDNF or proBDNF/BDNF ratio, in MDD were lower than those in healthy controls. Fluvoxamine however did not change serum levels of BDNF, proBDNF, and proBDNF/BDNF ratio at least within 4 weeks. Finally, no correlations exist between plasma levels of fluvoxamine and the changes in the HAMD17 scores or serum BDNF levels. In short, there is no association between serum levels of proBDNF, BDNF, or proBDNF/BDNF ratio and fluvoxamine response in MDD patients at least within 4 weeks of treatment. Using a different antidepressant medication on proBDNF/BDNF could be useful to determine the specificity of the effect of fluvoxamine.

## Competing interests

Professor Nakamura has received grant support from Dainippon-Sumitomo Pharma Co., Tanabe-Mitsubishi Pharma Co., and Astellas Pharma Co., Ltd in 2013. The other authors declare that they have no competing interests.

## Authors’ contributions

RY designed the study, measured the serum BDNF, proBDNF, and plasma fluvoxamine, wrote the first draft, and managed the literature searches. TK performed the statistical analyses. HH, KA, AK, WU-N, and AI-S collected the clinical data. NI and JN wrote the final manuscript. All of the authors took part in either drafting the article or revising it critically for important intellectual content and approved the final manuscript.
